# The Liver

**Published:** 1892-08

**Authors:** 


### THE LIVER.

BY A DOCTOR IN GOOD HEALTH.

Probably no organ in the body is so much medicated as the liver, and between bad diet and bad medicine, it has a pretty hard time of it.

Among patent nostrums, those for the liver outnumber all others about two to one ; their name is legion. What do medicines which are supposed to help the liver do ? They do nothing, but the liver is forced to do something with the medicines. When a person takes an emetic, we say it acts upon the stomach, when, in fact, the stomach acts upon the medicine. Suppose a reptile entered a room, and the persons present drove it out ; would we say that it was the reptile that acted? It is the same when the stomach, or the liver, or any other organ of the body, finds some intruding substance likely to work mischief ; it goes to work to drive it out, or dispose of it somehow. A liver medicine is one which comes within the province of the liver to dispose of. It is a liver whip. We talk as if medicines had brains and intelligence to hunt up certain organs or tissues, and fix them over. The organ which is the most capable of getting rid of the foreign substance, is the one which is said to be acted upon. If it is something that the stomach cannot act upon, very likely it will pass into the domain of the liver.

What is the philosophy of liver medicines? They help the liver just as a whip helps a tired horse. Take a liver which is worn out and exhausted with too much hearty food, too much butter, fats and sweets and it needs just what a tired horse needs—rest, and proper food and drink. Lighten its load instead of putting on the whip. But the ordinary treatment is to use the lash, and put on the brakes at the same time,—to increase the burden and use the goad. The result of this kind of treatment is premature exhaustion of the liver. The man who treats his horse in this way, must after a while hunt around for another horse ; and the man who constantly abuses his liver, will be hunting a new liver.

This principle applies to all classes of medicines, but very few of them really act upon the liver or are acted upon. For instance, there is that "giant remedy,"—mercury, blue mass or calomel—which most people suppose has a powerful effect upon the liver ; there is such a change in the individual who has taken it, and so much bile discharged, that they naturally think the liver has been helped. Dr. Bennett, of Edinburg, made some interesting experiments to ascertain the real influence of mercury upon the liver, using a dog for a subject. The microscope shows very little difference between the liver of a dog and that of a human being, and he reasoned that if mercury would touch up the liver of a man, it would touch up the liver of a dog, since the functions are the same in each. He performed an operation upon the animal so that the bile was discharged outside the body through a silver tube, instead of taking its natural course. The quantity of bile secreted was weighed, measured and evaporated daily. When the dog recovered his usual health, and nature had adapted herself to the new order of things, mercury was given in varying doses, and it was found that its influence invariably was to diminish the amount of bile secreted instead of increasing it. This experiment was repeated numerously by Dr. Bennett and afterward by other physicians, and always with the same result. Still, calomel continues its hold with many; and one professor of a medical college in New York, delared that he would use calomel when the liver needed touching up, "in spite of Dr. Bennett and all the dogs in Edinburg."

How is it, we may ask, that there seems to be so much apparent effect from the use of liver medicines in the discharge of the bile? The explanation is this. The bile is not simply an excretion; its chief office is to help digest the food. It is poured out in the upper part of the alimentary canal, where it helps digest the fats, stimulates peristaltic action, preserves the food while digestion and absorption are going on, and other work previously explained. The amount of bile has been variously estimated at from three to nine pints daily; the average will probably be four or five pints every twenty-four hours. The most of it is re-absorbed, by going into the portal circulation, and so is ready for the next day's use. It does not go into the general circulation at all. Now, when a person takes mercury, the effect is to so poison the bile that it is not absorbable, and so instead of being used again, it is hurried out of the body as waste material. Thus although the actual amount of bile secreted is less, the amount discharged is greater. That is why mercury seems to make the liver act. This is true of some other liver medicines. But there are some medicines which do excite the action of the liver; salts are the very best whips of this kind, but they are nothing but whips.

But suppose we want to increase the activity of the liver, what shall we do? Here is a horse struggling up a steep hill with a heavy load; would a whip be the best thing for the horse? would it not be better to lighten the load? The analogy holds good with reference to the liver. Lighten its burdens of fat, sugar, condiments and big dinners, and encourage it with plenty of hot water, which will dilute the bile, and cause it to move more rapidly along its course and do its work easier. If a person is suffering from the immediate effects of over eating, and the alimentary canal is full of germs, the best thing is a dose of salts, to relieve the alimentary canal; but it will be of no real help to the liver. Increasing the supply of water in the system is increasing the pressure upon the blood vessels, and this will help to drive the waste material out, and save the liver much work.

